# Forced-Air Warming Temperature Settings for Treating Postoperative Hypothermia in the Postanesthesia Care Unit: Randomized Controlled Trial

**DOI:** 10.2196/85045

**Published:** 2026-01-28

**Authors:** Koravee Pasutharnchat, Rattaphol Seangrung, Sirikarn Sirisophaphong, Wilailuck Wongkum

**Affiliations:** 1 Department of Anesthesiology, Faculty of Medicine, Ramathibodi Hospital, Mahidol University, 270 Rama 6 Road, Ratchathewi, Bangkok, 10400, Thailand, 66 22011523, 66 22011569

**Keywords:** postoperative hypothermia, forced-air warming, effectiveness, postanesthesia care unit, temperature settings

## Abstract

**Background:**

Hypothermia, defined as a core body temperature below 36 °C, is a common postoperative complication associated with adverse outcomes, including delayed wound healing, infections, and increased bleeding.

**Objective:**

This randomized controlled trial evaluated the efficacy of different forced-air warming system temperature settings in treating postoperative hypothermia in the postanesthesia care unit.

**Methods:**

A total of 132 patients undergoing elective surgery at Ramathibodi Hospital between April 2023 and May 2024 were randomized into 3 groups (n=44 per group): group C (warming set to 38 °C), group F1 (warming set to 43 °C), and group F2 (warming set to 43 °C, reduced to 38 °C after achieving 36 °C)*.* Tympanic temperature was recorded at 5-minute intervals during rewarming and every 10 minutes after normothermia (≥36 °C) was achieved. The primary outcome was rewarming time. Secondary outcomes included the incidence of temperature drops, hemodynamic parameters, adverse events, and patient comfort scores.

**Results:**

Baseline characteristics and clinical variables, including vital signs, were comparable among groups (*P*>.05). Group F2 achieved the shortest mean rewarming time of 33.3 (SD 13.81) min; however, differences between groups were not statistically significant (*P*=.460). Group F2 had the lowest incidence of temperature drops below 36 °C after normothermia (1/44, 2.27%; *P*=.009). Group C had the highest incidence of rewarming exceeding 1 hour (10/44, 22.73%; *P*=.017).

**Conclusions:**

While rewarming times were similar across groups, the protocol using an initial setting of 43 °C followed by a reduction to 38 °C (group F2) effectively minimized temperature drops after normothermia, suggesting its superiority for managing postoperative hypothermia in the postanesthesia care unit.

## Introduction

Hypothermia, defined as a core body temperature below 36 °C, is a frequent complication in patients undergoing elective surgery [1]. Intraoperative hypothermia, if uncorrected, often leads to postoperative hypothermia, particularly in the recovery room, where insufficient warming measures can exacerbate the condition. The prevalence of postoperative hypothermia in the postanesthesia care unit (PACU) has been reported to range from 20% to 28% at arrival and from 18.5% to 26% within 30 minutes after arrival [2]. Postoperative hypothermia is clinically significant, as it has been associated with impaired wound healing, increased risk of surgical site infection, greater blood loss, cardiac arrhythmias, and prolonged hospitalization [3,4]. These adverse consequences highlight the importance of effective temperature management strategies throughout the perioperative period. Recent guidelines and reviews, including Enhanced Recovery After Surgery pathways and the clinical recommendations from the Royal College of Anesthesiologists of Thailand, emphasize the critical role of maintaining normothermia as a core component to reduce surgical site infections and hospital stay [5-8].

Active warming techniques, particularly forced-air warming (FAW), are widely implemented to reduce the incidence of perioperative hypothermia. FAW devices deliver warmed air (32 °C‐47 °C) through a specialized blanket, with built-in safety mechanisms to prevent overheating [9,10]. Systematic reviews have demonstrated that FAW is superior to conventional blankets, reducing the time to restore normothermia by more than an hour [11]. While these findings confirm its effectiveness in facilitating rewarming, the literature remains inconclusive regarding the optimal temperature setting for postoperative use. Most previous studies have focused on preoperative or intraoperative warming [12-18], whereas evidence for postoperative FAW application remains limited. Xu et al [19] reported that FAW at 42 °C was more effective than at 38 °C or conventional blankets in elderly patients undergoing joint replacement. However, the generalizability of that study was restricted by the narrow patient population, short operative times, and limited assessment of adverse events.

At our institution, the prevalence of postoperative hypothermia has remained notable despite the routine availability of FAW systems. Pisitsak et al [20] documented hypothermia in 20% of patients under regional anesthesia and in 16% under general anesthesia. More recent institutional data from 2019 to 2022 indicate an incidence of 23% among surgical patients recovering in the PACU. Furthermore, between 2022 and 2024, the prevalence of hypothermia ranged from 10.8% to 13.8% despite widespread FAW use across multiple surgical specialties, including general surgery, orthopedics, otolaryngology, obstetrics and gynecology, and cardiac surgery (Department of Anesthesiology, Faculty of Medicine Ramathibodi Hospital, Mahidol University. Internal statistical data analyzed via Power BI dashboard, unpublished data, January 2025). These findings suggest that, in addition to patient- and procedure-related factors, variability in FAW temperature settings contributes to inconsistent outcomes.

 Current practice in our PACUs uses FAW with adjustable temperature settings ranging from 38 °C to 43 °C; however, no standardized protocol exists to guide optimal temperature selection. This variability reflects broader uncertainty regarding the most effective strategy for postoperative rewarming and underscores the need for evidence-based guidance. To our knowledge, no prior randomized trial has evaluated a step-down temperature protocol (43 °C to 38 °C) in a mixed adult surgical population. By addressing this gap, the present study examines the effectiveness of different FAW temperature settings to inform a pragmatic and standardized PACU warming approach, with the goal of improving consistency in clinical practice and enhancing patient safety.

## Methods

### Study Design

This study was designed as a prospective randomized controlled trial.

### Patients

A total of 132 patients scheduled for elective surgery across various specialties, including general surgery, orthopedics, urology, otolaryngology, obstetrics and gynecology, and cardiac surgery, were enrolled between April 2023 and May 2024. The inclusion criteria consisted of patients aged 18 to 80 years, American Society of Anesthesiologists (ASA) physical status I to III, who were undergoing elective procedures under either general or regional anesthesia, with an expected operating time of at least 2 hours.

Exclusion criteria included patients with a core temperature exceeding 37.5 °C, evidence of infection (eg, sepsis), conditions precluding the use of forced-air warming (eg, burns, agitation, or delirium), those unable to communicate or complete the trial questionnaire, and patients who declined participation.

### Sample Size Calculation

A priori sample size calculation was conducted to ensure adequate statistical power for the study’s primary outcome: the duration of forced-air warming required for a patient’s core temperature to reach ≥36 °C. Based on a previous randomized controlled trial by Xu et al [19], utilizing a 2-sided significance level (α=.05), adjusted for multiple comparisons among the 3 groups (α/3=.017), corresponding to a *z* score of 2.41, a statistical power of 80% (*z* for β=0.84), an estimated SD of 6.45 minutes, and a clinically meaningful difference in rewarming time of 5 minutes, the calculation determined that 36 participants were required per group. To accommodate an anticipated 20% participant dropout rate, the sample size was prudently inflated to 44 participants for each of the 3 intervention groups. This resulted in a total sample size of 132 participants, ensuring robust statistical inference for our findings.

### Randomization

Randomization was performed using stratified block randomization with proportional allocation based on the type of anesthesia (general vs regional) to ensure balanced distribution of thermoregulatory impairment mechanisms across groups. A research assistant not involved in patient recruitment generated the computer-based random sequence using permuted blocks of variable size. Allocation was concealed using sequentially numbered, sealed opaque envelopes, which were opened only after participant enrollment. The study personnel responsible for enrollment were different from those assigning participants to groups to ensure the integrity of allocation concealment.

### Rewarming

Intraoperative management followed our institution’s standard of care, which included the routine use of fluid warmers, application of forced-air warming blankets, and continuous core temperature monitoring for all patients. Upon arrival at the PACUs, patients who met the preliminary criteria were assessed. Only those with a core temperature lower than 36 °C were enrolled and randomly allocated into 3 groups (n=44 per group): group C (forced-air warming set to 38 °C), group F1 (forced-air warming set to 43 °C), and group F2 (forced-air warming initially set to 43 °C, then reduced to 38 °C once the core temperature reached 36 °C).

All participants received identical warming systems and core temperature monitoring devices at the PACU. Rewarming was carried out using a forced-air warming system (Bair Hugger) with a blanket and a core temperature measurement device (Braun ThermoScan ear thermometer).

The rewarming process was monitored and recorded every 5 minutes during the active warming phase. In groups C and F1, the forced-air warmer was discontinued once the core temperature reached ≥36 °C, at which point patients were covered with a regular blanket and monitored every 10 minutes. In group F2, the setting was reduced to 38 °C upon reaching a core temperature of ≥36 °C, and patients were similarly monitored every 10 minutes until discharge from the PACU. Rewarming time was calculated as the time taken for the core temperature to rise from baseline to ≥36 °C, measured in minutes.

### Outcome Measures

The primary outcome was the rewarming time, defined as the duration from the initiation of rewarming to the recovery of normothermia (core temperature ≥36 °C). Additionally, the incidence of a decrease in core temperature after achieving normothermia was recorded in each group.

Secondary outcomes included the incidence of adverse events—such as hypotension, hypertension, arrhythmias, nausea or vomiting, pain, and shivering—and patient satisfaction. Patient satisfaction was evaluated using 2 validated instruments: the 5-point Patient Comfort Scale, which measures overall comfort and satisfaction, and the 7-point Thermal Comfort Scale, which assesses subjective thermal sensation ranging from –3 (cold) to +3 (hot), with 0 representing thermal neutrality.

### Data Collection

Preoperative and intraoperative data were collected, including patient demographics, surgical procedure, operative time, anesthetic technique, blood loss, and fluid and blood product administration. Upon PACU admission, core temperature was recorded every 5 minutes during the rewarming phase by trained PACU nurses. To ensure consistency, the same nurse performed all assessments for a given patient using the same device and the same ipsilateral ear. Blinding of these nurses was not feasible because the FAW device displayed temperature settings during operation; consequently, the nurses were aware of group allocation, although patients remained blinded. Once the core temperature reached ≥36 °C, measurements continued every 10 minutes until discharge based on the Modified Aldrete scoring system. Throughout the PACU stay, adverse events were monitored continuously, and patient comfort and thermal comfort scores were assessed by the nurses at the time of discharge.

### Statistical Analysis

Data were analyzed using SPSS software version 27 (IBM Corp.). Continuous variables were expressed as mean (SD) or median (IQR), depending on the distribution, which was assessed using the Shapiro-Wilk test. Categorical variables were presented as counts and percentages.

For comparisons between groups, one-way ANOVA was used for normally distributed continuous variables, while the Kruskal-Wallis test was applied to non-normally distributed data. Post hoc analyses were performed using Tukey honest significant difference test for ANOVA and Dunn test for the Kruskal-Wallis test, as appropriate. Categorical variables were compared using the chi-square test or Fisher exact test, as required.

Monte Carlo simulation was utilized for the Fisher exact test extension in contingency tables larger than 2×2 where cell counts were sparse (expected count <5), ensuring robust *P* value estimation without violating asymptotic assumptions. Relative risks with 95% CI were reported for significant categorical outcomes. For continuous variables, effect sizes were expressed as Cohen *d* to ensure consistency and enhance clinical interpretability. This was an intention-to-treat analysis, and all randomized patients were analyzed in their assigned groups. A *P* value of <.05 was considered statistically significant.

### Ethical Considerations

This study was approved by the Human Research Ethics Committees of Ramathibodi Hospital, Mahidol University (approval number MURA2023/202) and registered at Thaiclinicaltrials.org on March 14, 2023 (approval number TCTR20231012004). Written informed consent was obtained from all participants before enrollment. Participants’ privacy and confidentiality were strictly protected, and all data were deidentified before analysis. No financial compensation was provided to participants for their participation in the study.

## Results

### Baseline Characteristics

**Figure 1.**
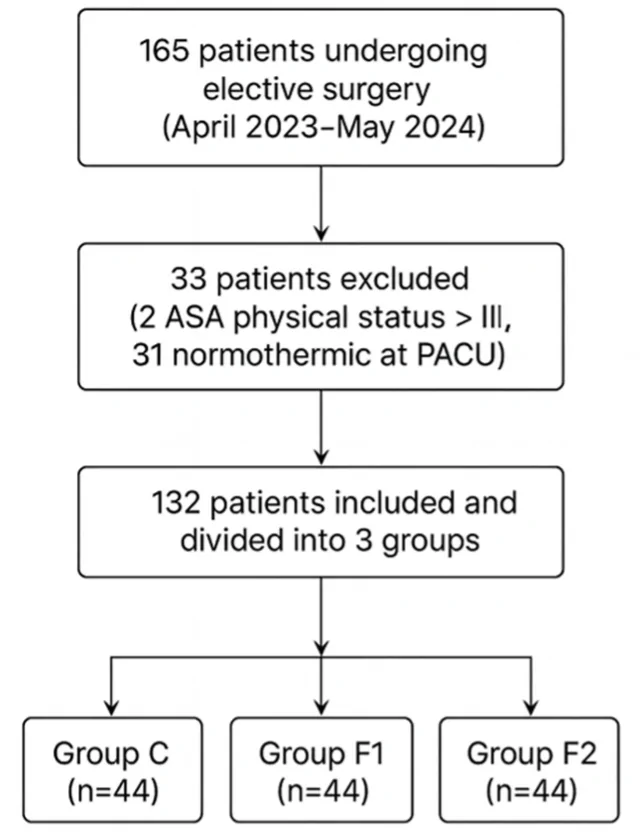
Flow diagram of patient enrollment. This diagram shows the screening and allocation process for all patients. ASA: American Society of Anesthesiologists; PACU: postanesthesia care unit.

A total of 165 patients were assessed for eligibility between April 2023 and May 2024. Thirty-three patients were excluded (2 with ASA physical status >III and 31 normothermic at PACU admission). Finally, 132 patients were included and equally divided into 3 groups (Figure 1): group C (rewarming set to 38 °C, n=44), group F1 (rewarming set to 43 °C, n=44), and group F2 (rewarming set to 43 °C until reaching a core temperature of 36 °C, then reduced to 38 °C, n=44). The baseline characteristics, including age, gender, ASA physical status, BMI, and underlying diseases, were comparable across all groups, as detailed in Table 1.

**Table 1.** Baseline characteristics of patients in the 3 groups.

Characteristic	Group C (n=44)	Group F1 (n=44)	Group F2 (n=44)	*P* value
Age, n (%)				.265
Elderly (age **≥**65 y)	10 (22.73)	13 (29.55)	17 (38.64)	
Nonelderly (age <65 y)	34 (77.27)	31 (70.45)	27 (61.36)	
Gender (male/female)				.083
Male	17	18	9	
Female	27	26	35	
ASA^a^ Physical status, n (%)				.965
>2	13 (29.55)	13 (29.55)	14 (31.82)	
≤2	31 (70.45)	31 (70.45)	30 (68.18)	
BMI (kg/m^2^), median (IQR)	23.85 (22‐26.95)	24 (22.43‐26.3)	23.67 (21.05‐27)	.738
Underlying diseases, n (%)				
Diabetes mellitus	3 (6.82)	8 (18.18)	7 (15.91)	.259
Hypertension	14 (31.82)	18 (40.91)	19 (43.18)	.511
Obesity	5 (11.36)	6 (13.64)	4 (9.08)	.798
Extreme age	10 (22.73)	12 (27.27)	15 (34.09)	.490
Heart disease	3 (6.82)	4 (9.09)	5 (11.36)	.927
Cerebrovascular disease	3 (6.82)	5 (11.36)	1 (2.27)	.295
Chronic kidney disease	4 (9.09)	5 (11.36)	2 (4.55)	.622
Cancer	5 (11.36)	8 (18.18)	11 (25)	.253
Respiratory disease	2 (4.55)	3 (6.82)	2 (4.55)	>.999
Others	11 (25)	14 (31.82)	7 (15.91)	.217

a ASA: American Society of Anesthesiologists.

### Intraoperative Data

No significant differences were observed among the 3 groups regarding operative time, type of operation, anesthetic technique, estimated blood loss, total fluid administration, total blood components used, or recorded temperatures, as summarized in Table 2.

**Table 2.** Intraoperative parameters of patients in the 3 groups.

Parameter	Group C (n=44)	Group F1 (n=44)	Group F2 (n=44)	*P* value
Operative time (min), median (IQR)	165 (120‐221.25)	180 (123.75‐223.75)	195 (137.5‐233.75)	.153
Anesthetic technique, n (%)				.926
Regional	15 (34.09)	14 (31.82)	14 (31.82)	
General	22 (50)	21 (47.73)	24 (54.55)	
Combined	7 (15.91)	9 (20.45)	6 (13.64)	
Operation, n (%)				.731
Open orthopedic surgery	18 (40.91)	18 (40.91)	17 (38.64)	
Arthroscopic/laparoscopic orthopedic surgery	8 (18.18)	7 (15.91)	11 (25)	
Open gynecologic surgery	3 (6.82)	4 (9.09)	4 (9.09)	
Laparoscopic gynecologic surgery	6 (13.64)	3 (6.82)	8 (18.18)	
Breast surgery	3 (6.82)	0 (0)	2 (4.55)	
Open general surgery	2 (4.55)	2 (4.55)	1 (2.27)	
Urological surgery	1 (2.27)	1 (2.27)	0 (0)	
Thoracic surgery	1 (2.27)	1 (2.27)	0 (0)	
Plastic surgery	18 (40.91)	1 (2.27)	0 (0)	
Vascular surgery	0 (0)	2 (4.55)	0 (0)	
Laparoscopic general surgery	2 (4.55)	4 (9.09)	1 (2.27)	
Otolaryngologic surgery	0 (0)	1 (2.27)	0 (0)	
Intraoperative period				
Large estimated blood loss (≥500 ml), n (%)	1 (2.27)	3 (6.82)	1 (2.27)	.435
Total fluid administered (ml), median (IQR)	975 (762.5‐1425)	900 (612.5‐1387.5)	1000 (712.5‐1487.5)	.808
Total blood component (ml), median (IQR)	0 (0)	0 (0)	0 (0)	.173
Temperature recorded (°C), mean (SD)	36.07 (0.43)	35.93 (0.37)	36.1 (0.32)	.139

### Postoperative Data

Postoperative outcomes recorded in the PACU are summarized in Table 3. No significant differences were observed among the 3 groups regarding tympanic temperature upon arrival or hemodynamic parameters, including blood pressure, heart rate, respiratory rate, and SpO_2_ (*P*>.05). However, a statistically significant difference was observed in the duration of PACU stay (*P*=.015). Post hoc comparisons revealed significant differences between group C versus group F2 and group F1 versus group F2, indicating a more favorable distribution of discharge times in group F2. Regarding electrocardiogram findings, adverse events were rare, with only 1 patient exhibiting bradycardia.

**Table 3.** Postoperative outcomes of patients in the 3 groups.

Parameter	Group C (n=44)	Group F1 (n=44)	Group F2 (n=44)	*P* value
Duration in PACU^a^ (min),^c^ median (IQR)	60 (60‐65)	60 (60‐60)	60 (60‐60)	.015
Tympanic temperature <36 °C upon arrival in the PACU, n (%)				.165
<35 °C	0 (0)	1 (2.27)	3 (6.82)	
≥35 °C	44 (100)	43 (97.73)	41 (93.18)	
Systolic blood pressure (mmHg), mean (SD)	134.07 (22.57)	134.53 (18.21)	135.95 (21.86)	.910
Diastolic blood pressure (mmHg), mean (SD)	76.32 (31.85)	79.09 (12.5)	94.45 (106.34)	.341
Respiratory rate (per min), mean (SD)	17.07 (3.25)	18.09 (3.2)	17.95 (3.43)	.290
Heart rate (per min), mean (SD)	68.61 (12.12)	72.55 (13.15)	74.75 (13.31)	.081
SpO_2_^b^ (%), median (IQR)	100 (99‐100)	100 (99‐100)	100 (99‐100)	.688

a PACU: postanesthesia care unit.

b SpO_2_: peripheral capillary oxygen saturation.

c A statistically significant difference was observed only in the duration of stay in PACU (*P*=.015; post hoc comparisons revealed significant differences for group C vs group F2 [Cohen *d*=6.29] and group F1 vs group F2 [Cohen *d*=8.55]).

### Effect of Different Rewarming Methods

**Figure 2.**
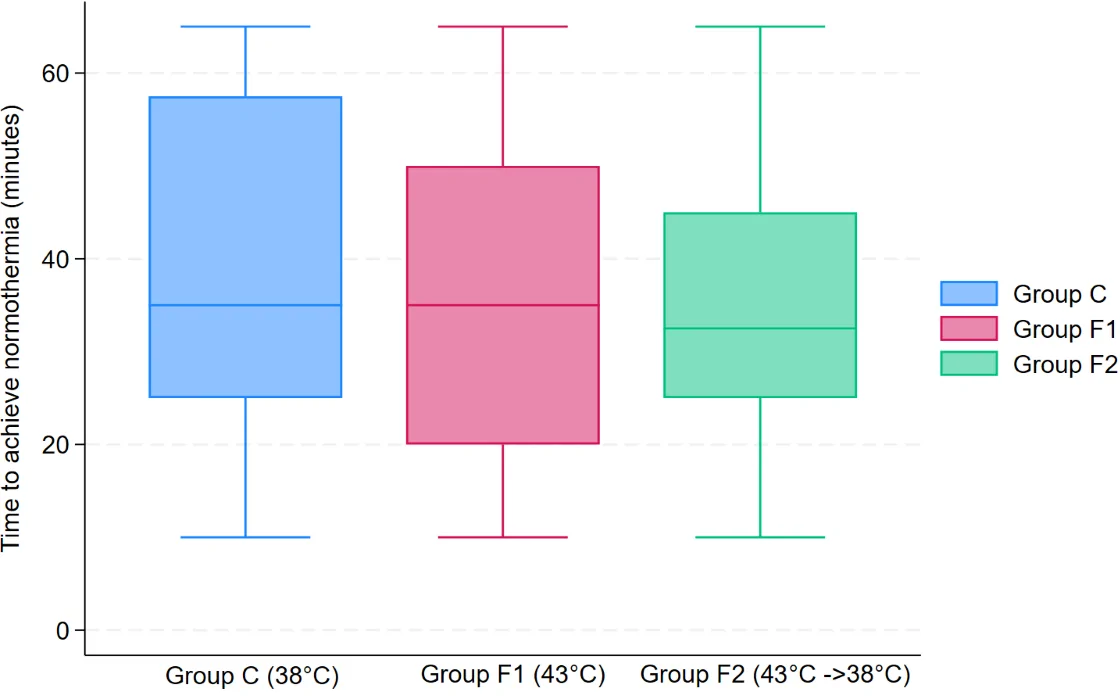
Comparison of rewarming outcomes among the 3 study groups. Box plot showing the distribution of time to achieve normothermia. The horizontal line within each box represents the median rewarming time. The top and bottom boundaries of the boxes indicate the IQR, and the whiskers extend to the minimum and maximum values. No statistically significant differences were observed (*P*=.460). Control group (group C): forced-air warming at 38 °C; group F1: forced-air warming at 43 °C; group F2: forced-air warming initially set at 43 °C, then reduced to 38 °C upon reaching 36 °C.

**Figure 3.**
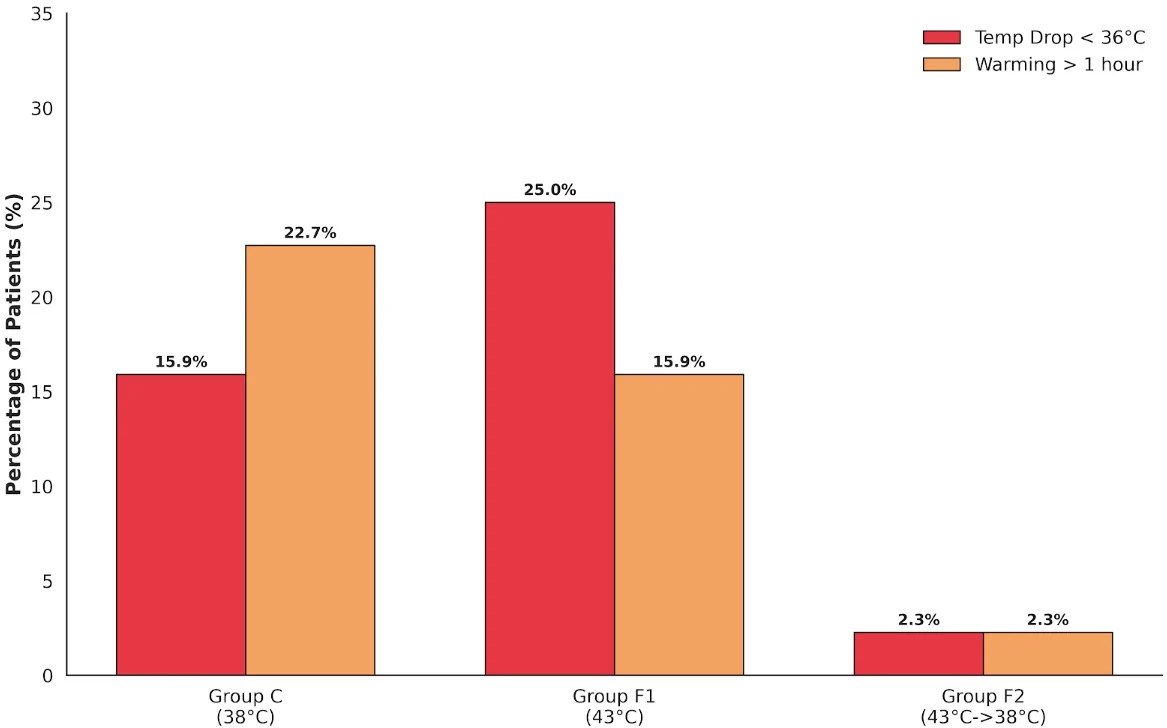
Comparison of rewarming outcomes among the 3 study groups. Clustered bar chart illustrating the incidence of recurrent hypothermia (core temperature dropping <36 °C after achieving normothermia) and the proportion of patients requiring active warming for more than 1 h. Group F2 demonstrated significantly lower rates for both outcomes compared to groups C and F1 (*P*=.009 and *P*=.017, respectively). Control group (group C): forced-air warming at 38 °C; group F1: forced-air warming at 43 °C; group F2: forced-air warming initially set at 43 °C, then reduced to 38 °C upon reaching 36 °C.

Rewarming outcomes across all dimensions are summarized in Table 4. The time to achieve normothermia is illustrated in Figure 2. Figure 3 shows the number of patients who experienced a drop in core temperature below 36 °C after achieving normothermia, and those who required rewarming for more than 1 hour.

No significant differences were observed among the 3 groups in hemodynamic parameters recorded in the PACU, including systolic and diastolic blood pressure, respiratory rate, heart rate, peripheral capillary oxygen saturation, and electrocardiogram findings (*P*>.05).

While the rewarming time did not differ significantly among the groups, the incidence of patients experiencing a drop in core temperature below 36 °C after achieving normothermia was significantly lower in group F2 compared to groups C and F1 (*P*=.009, Table 4). Patients exhibiting temperature decline required extended thermal support to restore or maintain normothermia. Consequently, a significantly higher proportion of patients in groups C and F1 required active warming for more than 1 hour compared to group F2 (*P*=.017). However, this prolonged warming requirement did not lead to a clinically relevant delay in discharge, as the median duration of PACU stay remained 60 minutes across all groups (Table 3).

**Table 4.** Rewarming outcomes of patients in the 3 groups.

Parameter	Group C (n=44)	Group F1 (n=44)	Group F2 (n=44)	*P* value
Mean rewarming time^a^ (min), mean (SD)	37.39 (16.58)	35.11 (15.64)	33.30 (13.81)	.460
Decrease temperature below 36 °C after achieving normothermia, n (%)	7 (15.91)	11 (25)	1 (2.27)	.009
Warming more than 1 h, n (%)	10 (22.73)	7 (15.91)	1 (2.27)	.017

a For the mean rewarming time, no significant differences were observed among groups; the mean differences (95% CI) compared to group C were −2.28 (−9.03 to 4.47) for group F1 and −4.09 (−10.46 to 2.28) for group F2. However, a statistically significant difference was observed in the proportion of patients with temperature decrease below 36 °C (*P*=.009; significant pairwise differences were observed for group F1 vs group F2 [relative risk (RR)=11.00; 95% CI 1.48‐81.61]) and those requiring warming for more than 1 h (*P*=.017; significant pairwise differences were observed for group C vs group F2 [RR=10.00; 95% CI 1.34‐74.84]).

### Adverse Events

In terms of postoperative adverse events, no significant differences were observed among the 3 groups. Pain was the most frequently reported complication, affecting 22.7% (10/44) of patients in group C and 18.2% (8/44) in both group F1 and group F2 (*P*=.826). Nausea and vomiting occurred infrequently, with an incidence ranging from 2.3%(1/44) to 4.6%(2/44) across the groups (*P*>.999). Shivering was reported in 4.6% (2/44) of patients in group C, 6.8% (3/44) in group F1, and 2.3% (1/44) in group F2 (*P*=.871). Hemodynamic events were rare, comprising 1 case of hypertension in group C, no events in group F1, and 1 case each of hypotension and hypertension in group F2 (all *P*>.999). No patients experienced arrhythmia or other adverse effects. Overall, the incidence of postoperative complications was low and comparable among the groups, supporting the safety of forced-air warming across different temperature settings.

Patient comfort, as measured by satisfaction levels, showed a significant difference among the groups (*P*=.049), along with the average comfort scores (*P*=.039). The proportion of patients reporting being “very much satisfied” was 27.27% (12/44) in group C, 43.18% (19/44) in group F1, and 52.27% (23/44) in group F2 (Table 5). However, there was no significant difference in the thermal comfort scale among the groups (*P*=.131).

**Table 5.** Patient satisfaction in the 3 groups.

Parameter	Group C (n=44)	Group F1 (n=44)	Group F2 (n=44)	*P* value
Patient’s comfort, n (%)				.049
Very much satisfied	12 (27.27)	19 (43.18)	23 (52.27)	
Somewhat satisfied	29 (65.91)	21 (47.73)	21 (47.73)	
Undecided	2 (4.55)	4 (9.09)	0 (0)	
Not really satisfied	0 (0)	0 (0)	0 (0)
Not at all satisfied	1 (2.27)	0 (0)	0 (0)	
Patient’s comfort (average score), median (IQR)	4 (4-5)	4 (4-5)	5 (5-5)	.039
Thermal comfort scale, n (%)				.131
Hot	1 (2.27)	4 (9.09)	2 (4.55)	
Warm	34 (77.27)	32 (72.73)	31 (70.45)	
Slightly warm	6 (13.64)	2 (4.55)	9 (20.45)	
Neutral	2 (4.55)	6 (13.64)	2 (4.55)	
Slightly cold	1 (2.27)	0 (0)	0 (0)	
Cool	0 (0)	0 (0)	0 (0)
Cold	0 (0)	0 (0)	0 (0)
Thermal comfort scale (average score), median (IQR)	2.00(2-2)	2.00(2-2)	2.00 (1.25‐2)	.676

## Discussion

### Principal Findings

Postoperative hypothermia is a frequent complication of both general and regional anesthesia, primarily resulting from thermoregulatory impairment and internal heat redistribution [21]. While previous research identified FAW at 42 °C as effective for elderly patients [19], evidence regarding the optimal temperature setting for the general surgical population remains limited. Consequently, this trial aimed to evaluate the most effective and efficient rewarming protocol for patients undergoing various surgical procedures.

In this study, hypothermia was defined as a core temperature <36 °C upon PACU admission. Tympanic thermometry was selected over invasive nasopharyngeal or rectal probes, as used in previous studies [19,22], to prioritize patient comfort during the awake recovery phase.

The principal finding of this study is that increasing the FAW setting from 38 °C to 43 °C did not yield a statistically significant reduction in the overall rewarming time to normothermia*.* While group F2 achieved the target temperature approximately 3 to 4 minutes faster than the control group, the precision estimates provided by the 95% CI suggest that this difference is negligible. Given that all groups achieved normothermia within a comparable timeframe, the variation in rewarming speed appears to lack clinical relevance for PACU throughput.

Several physiological factors may explain why higher settings did not produce faster rewarming, a finding that contrasts with some previous studies [19]. Peripheral vasoconstriction can limit the rate of convective heat transfer from the skin to the core, creating a “plateau effect” regardless of the external heat gradient provided by higher FAW settings. Moreover, as the core temperature approaches the normal thermoregulatory threshold, the body initiates vasodilation to redistribute heat, preventing a linear increase in core temperature [21,23]. Device-specific factors, such as automatic safety regulation at higher settings or variability in blanket positioning, may have further minimized the actual difference in heat delivery.

Although rewarming rates were comparable, the group F2 protocol demonstrated superior thermal stability. Unlike group C, which exhibited a significantly higher incidence of prolonged rewarming, the step-down protocol (group F2) effectively minimized the number of “outliers”—patients requiring extended care due to thermal instability. The prolonged rewarming observed in group C is likely due to several physiological and thermodynamic factors. At a lower temperature (38 °C), the gradient between the patient’s core temperature and the surrounding warming environment is reduced, leading to a slower rate of heat transfer [24]. Additionally, peripheral vasoconstriction limits blood flow to the skin and extremities, impeding the transport of externally applied heat to the core [25]. Furthermore, the reduced metabolic rate associated with hypothermia decreases endogenous heat generation, collectively contributing to the extended recovery time [21]. Consequently, the requirement for prolonged active warming in groups C and F1 likely contributed to the statistical difference observed in the total duration of PACU stay (*P*=.015). Although the median stay was consistent at 60 minutes across all groups, the distribution of discharge times suggests that while group F2 may not shorten the mandatory minimum recovery time, it optimizes unit throughput by reducing the incidence of prolonged stays.

### Clinical Implications

Regarding safety and comfort, the incidence of adverse events—including pain, nausea, vomiting, hemodynamic changes, and shivering—did not differ significantly among the groups, and no severe adverse events were observed. These results align with the safety profile reported by Xu et al [19] However, regarding patient experience, group F2 reported higher satisfaction scores related to comfort during rewarming compared to groups C and F1. This suggests that an initial high-temperature setting effectively enhances thermal comfort, while the subsequent reduction prevents the discomfort associated with overheating.

Intraoperative factors—including ambient cooling, fluid administration, and anesthesia-induced thermoregulatory impairment—are known to significantly impact rewarming. In this study, potential confounding was minimized through a standardized intraoperative care protocol that included routine fluid and forced-air warming. Furthermore, randomization successfully balanced these physiological stressors across study arms; as shown in Table 2, there were no significant differences in operative duration, total fluid volume, or anesthetic technique. Consequently, the observed differences in PACU outcomes can be primarily attributed to the specific postoperative warming protocols rather than intraoperative disparities.

### Strengths and Limitations

This study has notable strengths and limitations. A key strength is the rigorous randomization and standardized intraoperative care, which successfully balanced potential confounders such as operative duration and fluid volume across study arms. However, several limitations exist. First, strict environmental control of the PACU was challenging due to the open-plan nature of the unit. The ambient temperature fluctuated between 22 and 24 °C, which serves as a potential environmental confounder influencing convective heat loss. Nevertheless, this variation reflects real-world clinical conditions, potentially enhancing the ecological validity of our results. Second, regarding measurement, reliance on tympanic thermometry introduces inherent variability compared to the gold standard of invasive core monitoring. We acknowledge that readings can be affected by factors such as probe positioning, cerumen obstruction, and post-anesthetic peripheral vasoconstriction. To mitigate these inaccuracies, we strictly standardized the technique by using the same device and assessing the ipsilateral ear throughout the study, aiming to balance measurement precision with patient comfort in the awake state. Third, data collection involved intermittent recordings at 5-minute intervals rather than continuous electronic monitoring. While this frequency is clinically practical, it may lack the temporal resolution to capture rapid, transient temperature fluctuations during the active rewarming phase, potentially masking the true extent of thermal variability. Finally, we did not perform a formal cost-effectiveness analysis. Although the step-down protocol (group F2) showed potential for optimizing PACU throughput, future studies including economic evaluations are needed to confirm the financial implications of these warming strategies.

### Conclusions

In summary, although varying temperature settings of forced-air warming systems produced comparable rewarming times, the protocol involving an initial setting of 43 °C followed by a reduction to 38 °C (group F2) was associated with superior maintenance of normothermia and a significantly lower incidence of postoperative hypothermia recurrence*.* These findings underscore the potential benefits of implementing optimized warming protocols to enhance patient outcomes in the PACU.

## Abbreviations

ASA: American Society of Anesthesiologists
FAW: forced-air warming
PACU: postanesthesia care unit
