# Correction: Forced-Air Warming Temperature Settings for Treating Postoperative Hypothermia in the Postanesthesia Care Unit: Randomized Controlled Trial

**DOI:** 10.2196/110801

**Published:** 2026-10-27

**Authors:** Koravee Pasutharnchat, Rattaphol Seangrung, Sirikarn Sirisophaphong, Wilailuck Wongkum

**Affiliations:** 1 Department of Anesthesiology, Faculty of Medicine, Ramathibodi Hospital, Mahidol University, 270 Rama 6 Road, Ratchathewi, Bangkok, 10400, Thailand, 66 22011523, 66 22011569

 In “Forced-Air Warming Temperature Settings for Treating Postoperative Hypothermia in the Postanesthesia Care Unit: Randomized Controlled Trial” [1], the authors noted 9 errors. The following corrections have been made:

First, in the Methods section of the abstract, the following sentence was revised:

*A total of 132 patients undergoing elective surgery at Ramathibodi Hospital between April 2023 and May 2024 were randomized into 3 groups (n=44 per group): group C (warming set to 38 °C), group F1 (warming set to 42 °C), and group F2 (warming set to 42 °C, reduced to 38 °C after achieving 36 °C).*

The corrected sentence now reads:

*A total of 132 patients undergoing elective surgery at Ramathibodi Hospital between April 2023 and May 2024 were randomized into 3 groups (n=44 per group): group C (warming set to 38 °C), group F1 (warming set to 43 °C), and group F2 (warming set to 43 °C, reduced to 38 °C after achieving 36 °C).*

Second, in the Conclusions section of the abstract, the following sentence was revised:

*While rewarming times were similar across groups, the protocol using an initial setting of 42 °C followed by a reduction to 38 °C (group F2) effectively minimized temperature drops after normothermia, suggesting its superiority for managing postoperative hypothermia in the postanesthesia care unit.*

The corrected sentence now reads:

*While rewarming times were similar across groups, the protocol using an initial setting of 43 °C followed by a reduction to 38 °C (group F2) effectively minimized temperature drops after normothermia, suggesting its superiority for managing postoperative hypothermia in the postanesthesia care unit.*

Third, in the final paragraph of the Introduction, the following sentences were revised:

*Current practice in our PACUs uses FAW with adjustable temperature settings ranging from 38 °C to 42 °C; however, no standardized protocol exists to guide optimal temperature selection. This variability reflects broader uncertainty regarding the most effective strategy for postoperative rewarming and underscores the need for evidence-based guidance. To our knowledge, no prior randomized trial has evaluated a step-down temperature protocol (42 °C to 38 °C) in a mixed adult surgical population.*

The corrected sentences now read:

*Current practice in our PACUs uses FAW with adjustable temperature settings ranging from 38 °C to 43 °C; however, no standardized protocol exists to guide optimal temperature selection. This variability reflects broader uncertainty regarding the most effective strategy for postoperative rewarming and underscores the need for evidence-based guidance. To our knowledge, no prior randomized trial has evaluated a step-down temperature protocol (43 °C to 38 °C) in a mixed adult surgical population.*

Fourth, in the Rewarming section of the Methods, the following sentence was revised:

*Only those with a core temperature lower than 36 °C were enrolled and randomly allocated into 3 groups (n=44 per group): group C (forced-air warming set to 38 °C), group F1 (forced-air warming set to 42 °C), and group F2 (forced-air warming initially set to 42 °C, then reduced to 38 °C once the core temperature reached 36 °C).*

The corrected sentence now reads:

*Only those with a core temperature lower than 36 °C were enrolled and randomly allocated into 3 groups (n=44 per group): group C (forced-air warming set to 38 °C), group F1 (forced-air warming set to 43 °C), and group F2 (forced-air warming initially set to 43 °C, then reduced to 38 °C once the core temperature reached 36 °C).*

Fifth, in the Baseline Characteristics section of the Results, the following sentence was revised:

*Finally, 132 patients were included and equally divided into 3 groups (Figure 1): group C (rewarming set to 38 °C, n=44), group F1 (rewarming set to 42 °C, n=44), and group F2 (rewarming set to 42 °C until reaching a core temperature of 36 °C, then reduced to 38 °C, n=44).*

The corrected sentence now reads:

*Finally, 132 patients were included and equally divided into 3 groups (Figure 1): group C (rewarming set to 38 °C, n=44), group F1 (rewarming set to 43 °C, n=44), and group F2 (rewarming set to 43 °C until reaching a core temperature of 36 °C, then reduced to 38 °C, n=44).*

Sixth, in the originally published Figure 2, all labels within the figure image that referred to the high-temperature setting displayed 42 °C. The figure caption read:

*Control group (group C): forced-air warming at 38 °C; group F1: forced-air warming at 42 °C; group F2: forced-air warming initially set at 42 °C, then reduced to 38 °C upon reaching 36 °C.*

In the corrected Figure 2 (included here as Figure 1), all labels within the figure image that refer to the high-temperature setting display 43 °C. The corrected version of Figure 2 has replaced the originally published figure. The figure caption now reads:

*Control group (group C): forced-air warming at 38 °C; group F1: forced-air warming at 43 °C; group F2: forced-air warming initially set at 43 °C, then reduced to 38 °C upon reaching 36 °C.*

**Figure 1.**
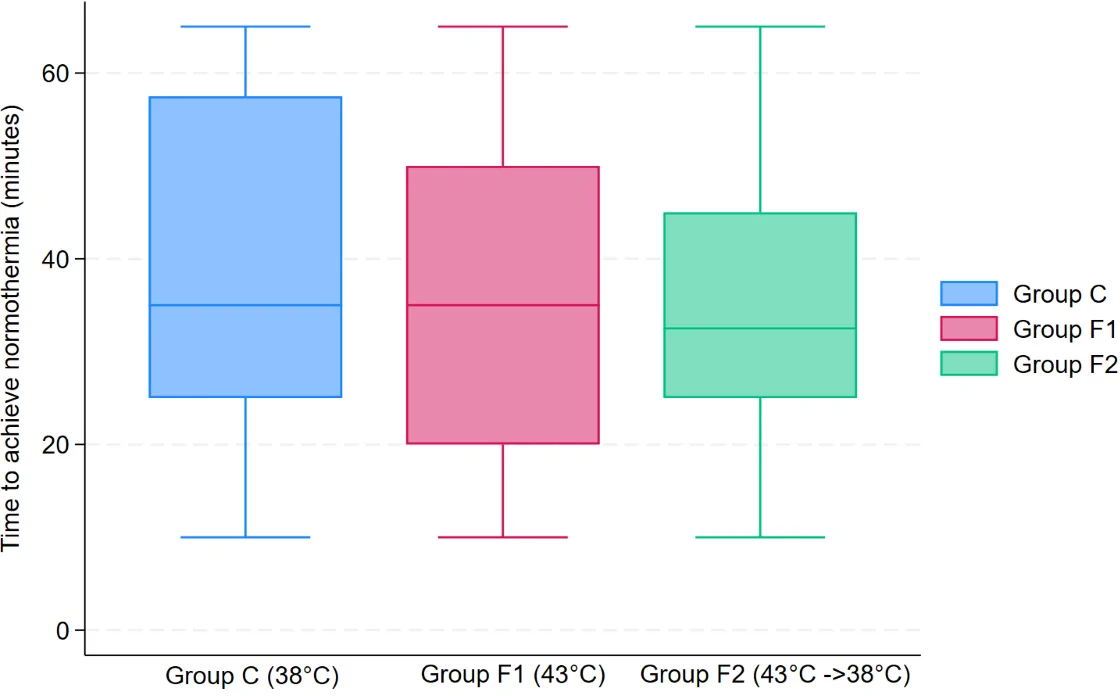
Revised version of Figure 2: Comparison of rewarming outcomes among the 3 study groups. Box plot showing the distribution of time to achieve normothermia. The horizontal line within each box represents the median rewarming time. The top and bottom boundaries of the boxes indicate the IQR, and the whiskers extend to the minimum and maximum values. No statistically significant differences were observed (*P*=.460). Control group (group C): forced-air warming at 38 °C; group F1: forced-air warming at 43 °C; group F2: forced-air warming initially set at 43 °C, then reduced to 38 °C upon reaching 36 °C.

Seventh, in the originally published Figure 3, all labels within the figure image that referred to the high-temperature setting displayed 42 °C. The figure caption read:

*Control group (group C): forced-air warming at 38 °C; group F1: forced-air warming at 42 °C; group F2: forced-air warming initially set at 42 °C, then reduced to 38 °C upon reaching 36 °C.*

In the corrected Figure 3 (included here as Figure 2), all labels within the figure image that refer to the high-temperature setting display 43 °C. The corrected version of Figure 3 has replaced the originally published figure. The figure caption reads:

*Control group (group C): forced-air warming at 38 °C; group F1: forced-air warming at 43 °C; group F2: forced-air warming initially set at 43 °C, then reduced to 38 °C upon reaching 36 °C.*

**Figure 2.**
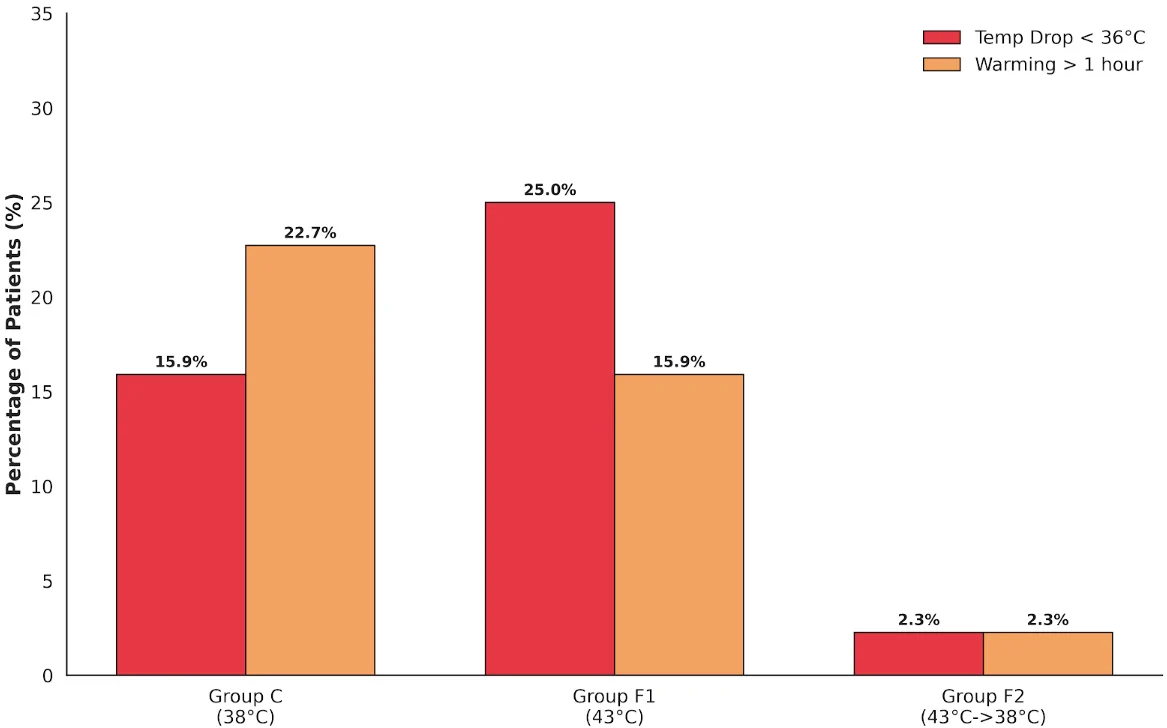
Revised version of Figure 3: Comparison of rewarming outcomes among the 3 study groups. Clustered bar chart illustrating the incidence of recurrent hypothermia (core temperature dropping <36 °C after achieving normothermia) and the proportion of patients requiring active warming for more than 1 h. Group F2 demonstrated significantly lower rates for both outcomes compared to groups C and F1 (*P*=.009 and *P*=.017, respectively). Control group (group C): forced-air warming at 38 °C; group F1: forced-air warming at 43 °C; group F2: forced-air warming initially set at 43 °C, then reduced to 38 °C upon reaching 36 °C.

Eighth, in the Principal Findings section of the Discussion, the following sentence was revised:

*The principal finding of this study is that increasing the FAW setting from 38 to 42 °C did not yield a statistically significant reduction in the overall rewarming time to normothermia.*

The corrected sentence now reads:

*The principal finding of this study is that increasing the FAW setting from 38 °C to 43 °C did not yield a statistically significant reduction in the overall rewarming time to normothermia.*

Finally, in the Conclusions section of the Discussion, the following sentence was revised:

*In summary, although varying temperature settings of forced-air warming systems produced comparable rewarming times, the protocol involving an initial setting of 42 °C followed by a reduction to 38 °C (group F2) was associated with superior maintenance of normothermia and a significantly lower incidence of postoperative hypothermia recurrence.*

The corrected sentence now reads:

*In summary, although varying temperature settings of forced-air warming systems produced comparable rewarming times, the protocol involving an initial setting of 43 °C followed by a reduction to 38 °C (group F2) was associated with superior maintenance of normothermia and a significantly lower incidence of postoperative hypothermia recurrence.*

The correction will appear in the online version of the paper on the JMIR Publications website together with the publication of this correction notice. Because this was made after submission to PubMed, PubMed Central, and other full-text repositories, the corrected article has also been resubmitted to those repositories.
